# Supplementary material for: Disentangling genetic and epigenetic determinants of ultrafast adaptation
Source: Mol Syst Biol. 2016 Dec 15;12(12):892. doi: 10.15252/msb.20166951 (PMC5199126; doi:10.15252/msb.20166951)
Supplement: Supplementary file 1 — Appendix [file MSB-12-892-s001.pdf]

# APPENDIX

## TABLE OF CONTENTS

Appendix Supplementary Figure legends S1-S8

Appendix Supplementary Figures S1-S8

Appendix Tables S1-S3

## APPENDIX SUPPLEMENTARY FIGURE LEGENDS

**Appendix Figure S1 Ultrafast adaptation to arsenic in a batch-to-batch experimental evolution regime**

A) Schema of the experimental evolution regime, in population genetics terms. A founder population of haploid yeast cells was derived by clonal expansion from a single founder cell. From the founder population, 72 experimental populations were initiated, each from an initial population size of $N \approx 10^5$. Populations corresponded to four repeats evolving in parallel to each of 18 environmental challenges. Populations were cultivated in batch mode (350 $\mu$L, microtitre format) and allowed to expand clonally until exhausting available resources and entering into stationary phase at $N \approx 3.5*10^6$ cells. $N \approx 10^5$ cells were randomly subsampled from each population by pipetting and re-inoculated in fresh, nutrient rich stress medium. The process was repeated for a total of 50 batch-to-batch cultivations (≈ 250 generations). Samples were extracted from each adapting population every 25$^{th}$ generation and frozen at -80C in glycerol. For fitness component extraction (see Methods) the frozen populations were revived and micro-cultivated ($n$ = 2) together with the founder ($n$ = 8), randomizing positions. B) Doubling time (log(2)) relative the founder of ($n$ = 4) independent populations (coloured lines) adapting to each of 19 environmental challenges. C) Number of viable cells, measured as the number of Colony Forming Units (CFU), in 1 mL of founder (WT) and *FPS1* mutated cell populations growing with and without 5mM As(III). For each type of sample, $n$ = 12 replicate populations initiated from equally sized clonal colonies and cultivated to OD = 0.5 - 1.5. The cell density was adjusted by dilution or concentration to OD = 1.00 and then diluted 1:10000. 50 $\mu$L of each dilution were evenly spread on solid agar medium plates without As(III). The number of colony forming units (CFU) was counted on each plate (100-200 colonies were observed per plate) after 3 days. CFU per mL in each population was calculated taking the dilution factor into account and a mean was calculated over all 12 replicates, for each type of sample. Error bars = S.E.M. As(III) exposure did not significantly (Student's t-test, $p$ > 0.05) affect viability in either founder or *FPS1* mutation carrying cells. We counted total cells by flow cytometry and estimated 1.2 (flow cytometry, $n$ = 1) and 0.9 - 1.1 (hemo cytometry, $n$ = 2) x $10^7$ cells per mL of OD = 1.00 medium. This may be a slight underestimation, as some cell aggregates are expected to resist sonication and be counted as one cell.

**Appendix Figure S2 Arsenic adaptive variants are neutral or negative in the absence of arsenic.**

Arsenic adaptive alleles (*ACR1, ACR2, ACR3, BET2* and *SWH1* duplication, *ask10* G680827A and *fps1* G51753T) were reconstructed in the founder (WT) background and micro-cultivated ($n$=8) in absence of arsenic. The lag, rate and efficiency of growth were extracted from high density growth curves, log(2) transformed and normalized to the corresponding mean ($n$ = 8) of founder (WT) populations cultivated on the same plate. Gene duplications were represented by insertions of centromeric plasmid with the gene on and were compared to WT cells with an empty plasmid. Statistically significant (Homoscedastic Student's t-test, FDR, $q$<0.01) deviations from the founder are indicated.

**Appendix Figure S3 Ultrafast arsenic adaptation associates with rapid fixation of *FPS1*, *ASK10* and *ACR3* mutations**

Adapting P1-P4 populations were sampled at every 25th generation and deep sequenced. The data is identical to that in Figure 2B, except that no filtering for SNPs predicted to affect protein function was performed. The frequency (left *y*-axis) of high quality SNPs with a total read depth of >100 (over all time-points) and frequency of >20% in ≥3 time points are shown. For the *ACR3* containing duplicated region in P2, the grand copy number mean of all the segments within the duplicated region (chr. XVI 880799 to 944600) is shown on the right *y*-axis. Color indicates variation. Bold line = causative mutations. Tolerated = SIFT > 0.05. Dubious = unlikely to encode a true ORF. Mito = mitochondrial. * = reconstructed *FPS1* mutation.

**Appendix Figure S4 Positive pleiotropy accelerates adaptation independent of adaptation speed**

Each dot corresponds to one simulated population. The 204 fastest scenarios, recovering 75% of unstressed performance in ≤100 cell divisions, are displayed. Patterns and conclusions are identical across all 500 simulated populations (data not shown). *y*-axis shows the relative increase in the speed of adaptation due to positive pleiotropy. The measure was calculated as the difference in adaptation speed between model M2 (both rate and lag affected, with equal effect size and direction) and model M1 (only rate affected) divided by model M1. *x*-xis show the speed of adaptation in each scenario. The speed of adaptation was expressed as the time to recover 25% (left panel), 50% (middle panel) and 75% (right panel) of the cell division time of an unstressed population. The black line shows a loess fit (span parameter 0.75) with a 95% confidence interval (grey area). Observe that the lines are close to horizontal. Thus, the adaptive benefits of positive pleiotropy are independent of adaptation speed.

**Appendix Figure S5 Selection coefficients for mutations driving arsenic adaptation**

A) Estimating selection coefficients for mutations driving ultrafast arsenic adaptation. A competition assay simulation followed the frequency of *ACR3* (left panel), *ASK10* (middle panel), and *FPS1* (right panel) mutations emerging in a single cell at $t = 0$ in population of WT cells exposed to 5 mM As(III). Mutation effect sizes equal empirical with only rate effects (dotted lines), only lag effects (dashed lines) or both lag and rate effects (full lines) considered. Simulation data correspond to the black lines in Figure 3C. *y*-axis shows the logarithm of the frequency of the mutation ($p$) relative to WT ($1 - p$), *x*-axis shows time in generations. Regression slopes correspond to selection coefficient estimates, $s$. Note that selection coefficient estimates may be conservative, as no selection on growth efficiency is assumed. B) Robustness analysis of selection coefficient estimates. The sensitivity of selection coefficients (estimated in A) to measurement errors in population lags and doubling times is shown. Note that the estimated noise (coefficient of variation over replicates) is in the range of 2-3% and that replicates were randomized over plate positions and compared to WT populations included in the same plate, avoiding bias. Our selection coefficient estimates are therefore very robust to measurement error in the realistic range.

**Appendix Figure S6 Mutation target sizes for arsenic adaptive variants in *FPS1* and *ASK10***

A-B) Mutational target sizes for adaptive variants in *FPS1* and *ASK10* were estimated based on the approximations that: only complete loss-of-function mutations in these loci contribute to ultrafast adaptation, that complete loss-of-function mutations are well reflected by all possible stop gain base changes and all nonsynonymous mutations in highly conserved positions, and that all complete loss-of-function mutations have phenotype contributions equalling empirical measurements. For simplicity we assumed no transition/transversion bias, such that for a given reference base-pair all three possible base changes were equally likely. Under this assumption the number of base changes can be converted to a target size in basepairs by dividing by 3. A) Number of base changes leading to gain of a stop codon before a given base-pair position, in *FPS1* and *ASK10* respectively. The number of stop-gain base changes increases in a linear manner for both genes adding up to a total of 254 for *FPS1* and 469 *ASK10*. The resulting target size for a stop gain mutation in the full length of the two genes is 85 (*FPS1*) and 156 (*ASK10*). B) Possible nonsynonymous mutations in conserved sites in *FPS1* and *ASK10*, with conservation defined by a flexible SIFT (Sorting Intolerant From Tolerant - http://sift-db.bii.a-star.edu.sg/public/Saccharomyces_cerevisiae/EF4.74/) score cut-off. With our chosen cut-off for a loss-of-function mutation (SIFT<0.06, corresponding to the highest SIFT score observed for a loss-of-function mutation in the arsenic adapted strains). There are 1675 (*FPS1*) and 2360 (*ASK10*) nonsynonymous base substitutions leading to loss-of-function, for a resulting target size of 558 (*FPS1*) and 787 (*ASK10*) respectively. Stop gain and loss-of-function nonsynonymous mutations are non-overlapping so the total target sizes are 643bp (*FPS1*) and 943bp (*ASK10*) respectively.

**Appendix Figure S7 Correspondence between our data and population genetics theory**

There is a close correspondence between existing theory of dynamics of adaptation in asexuals (black) reviewed by (Sniegowski & Gerrish, 2010) and our data (colors). **Right part (black lines and texts):** Sniegowski & Gerrish discusses a simplified case where beneficial mutations arise with mutation rate $\mu$ and a constant selection coefficient $s$ in a population with effective population size $N_e$. In this case the expected number of competing mutations is given by $2N_e\mu ln(N_e s/2)$ ($y$-axis) and the probability that a given beneficial mutation meets no competitors on its way to fixation is inversely related to this number. The adaptive landscape is divided into three regimes. In the *strong selection, weak mutation (SSWM) regime*, where $2N_e\mu ln(N_e s/2) << 1$, the supply of mutations is limiting adaptation speed and the population evolves via isolated adaptive substitution. At the other extreme, in the *weak selection, strong mutation (WSSM) regime*, where $2N_e\mu ln(N_e s/2) >> 1$, beneficial mutations are very common relative to their selection coefficient and adaptation speed is limited by the selection coefficients. Between these two regimes is the *strong selection, strong mutation (SSSM) regime* where the dynamics become complex, and the co-occurrence and interference between beneficial mutations only occur occasionally. Stochastic modelling is widely used to understand adaptation in this regime. **Left part (colored lines and dots):** In our experiments $N_e$ is around $5*10^5$ (the harmonic mean of population sizes over one batch cycle (Lenski et al, 1991) The estimated selection coefficients for the reconstructed *ASK10*, *FPS1* and *ACR3* mutations are in the range $0.36 - 0.64$. For the purpose of this figure the three mutations are

lumped into a single adaptive mutation with *s = 0.5* and the estimated beneficial mutation rate in each of the three genes is ~8.25x$10^{-7}$ mutations/division. With these basal parameter values (middle point on green dashed line) the expected number of competing mutations is ~10, placing our dataset in the *strong selection, strong mutation* regime. Increasing $\mu$ (*x*-axis) or $N_e$ (line color) brings us fast into the selection limited SSWM regime with hundreds of competing mutations and where adaptation speed is limited by the selection coefficient. The insensitivity of adaptation speed to increased mutation rate is reflected in Figure 3D. The speed of extinction of the wild-type drops markedly from 1x to 3x, but further increase (5x and 10x) does not increase the adaptation speed much. Decreasing $\mu$ or *Ne* brings us towards the mutation limited WSSM regime where adaptation speed is limited by the expected waiting time for beneficial mutations to occur, and we see this limiting effect in the slowest replicates in the basal mutation rate panel in Figure 3D. We see that which adaptation regime becomes operative is much more sensitive to variation in $N_e$ and $\mu$ compared to variation in *s* (line type), justifying the above lumping of the selection coefficients of the *ASK10*, *FPS1* and *ACR3* mutations.

**Appendix Figure S8 Accounting for the late fixation of *FPS1* mutations in *ACR3* and *ASK10* clones**

Unidentified causal mutations account for a larger fraction of adaptive gains in *ASK10* and *ACR3* populations than in *FPS1* populations. We may therefore, at later stages of adaptation, have underestimated the fitness of *ACR3* and *ASK10* containing clones relative to *FPS1* containing clones. This could account for the late emergence and fixation of *FPS1* mutations in *ACR* and *ASK10* containing clones, which is observed in simulations (Figure 2D) but not in empirical populations. To test this possibility we let the adaptive gains of *ASK10* and *ACR3* mutations approach that of *FPS1* mutations, in replicated ($n$ = 100) simulations that otherwise are identical to those in Figure 3D. Figure shows number of simulated populations that end up ($t$ = 250) with high frequency mutations ($p$ > 0.9) in *FPS1, ACR3* and/or *ASK10*, given that *ASK10* and *ACR3* containing clones have closed 25%, 50, and 75% (plot rows) of the empirical gap to *FPS1* mutations and given basal, 3x, 5x and 10x (plot columns) mutation rates. The figure shows that *FPS1* mutations are less prone to emerge and fixate in *ACR3* and *ASK10* containing clones, when their performance approaches that of *FPS1* mutations.

**APPENDIX REFERENCES**

Lenski RE, Rose MR, Simpson SC, Tadler SC (1991) Long-term experimental evolution in Escherichia coli. I. Adaptation and divergence during 2,000 generations. *Am Nat* **138:** 1315-1341

Sniegowski PD, Gerrish PJ (2010) Beneficial mutations and the dynamics of adaptation in asexual populations. *Philosophical transactions of the Royal Society of London Series B, Biological sciences* **365:** 1255-1263

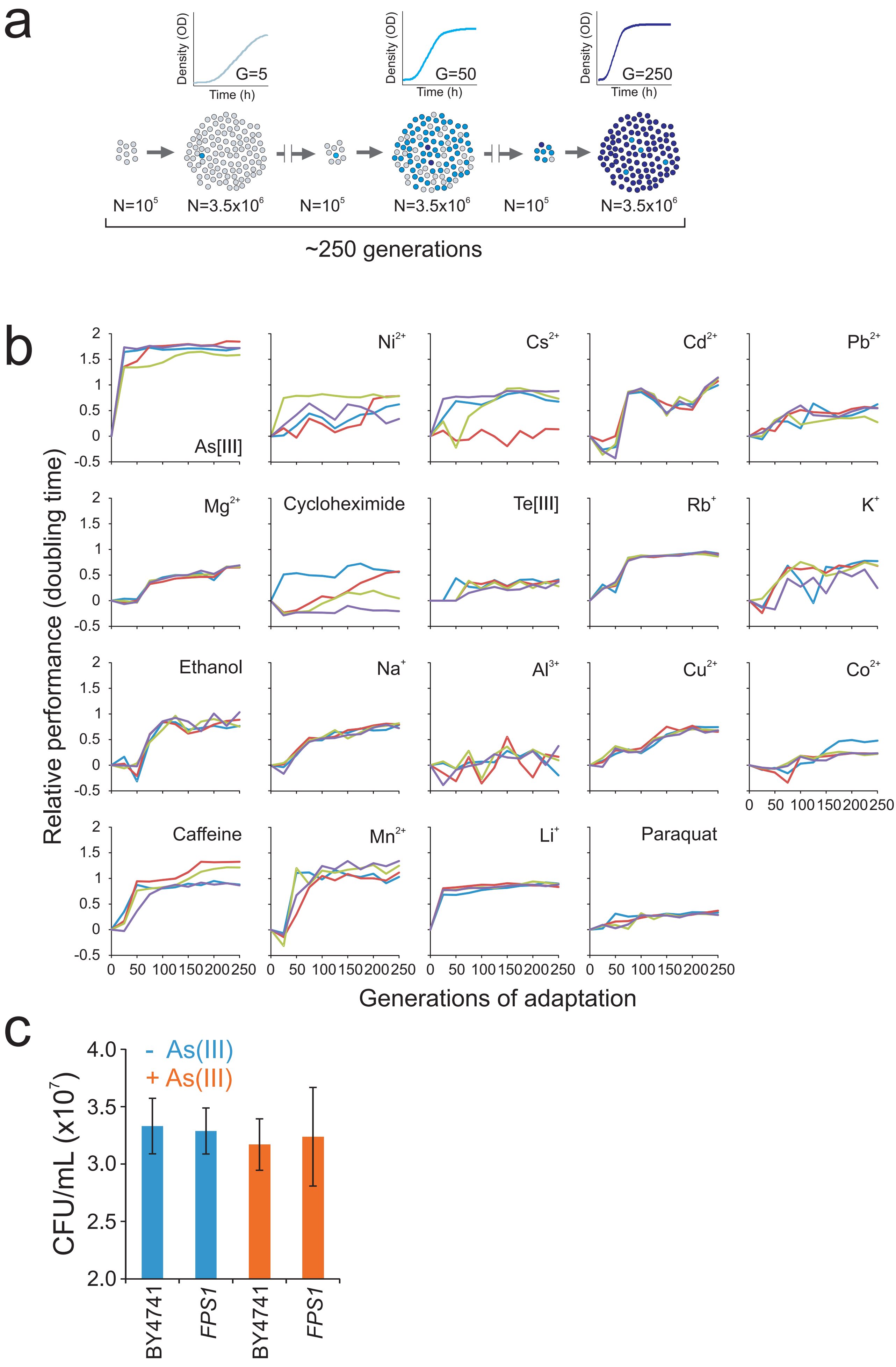


Figure S1 Gjuvsland et al

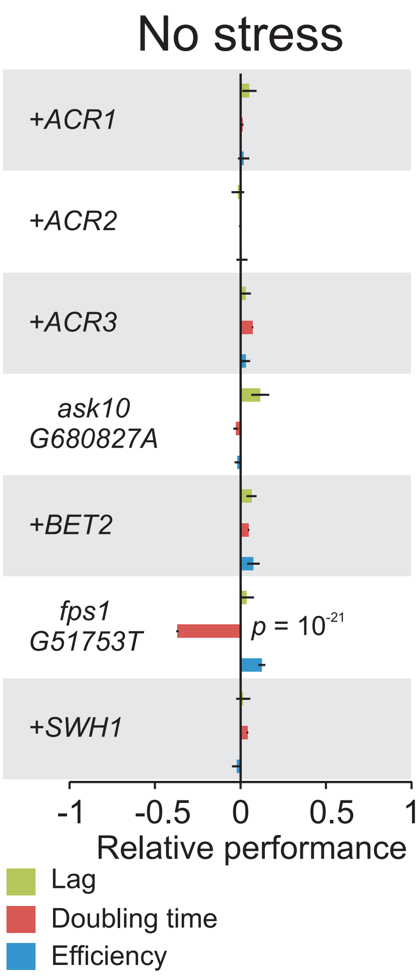


Figure S2 Gjuvsland et al

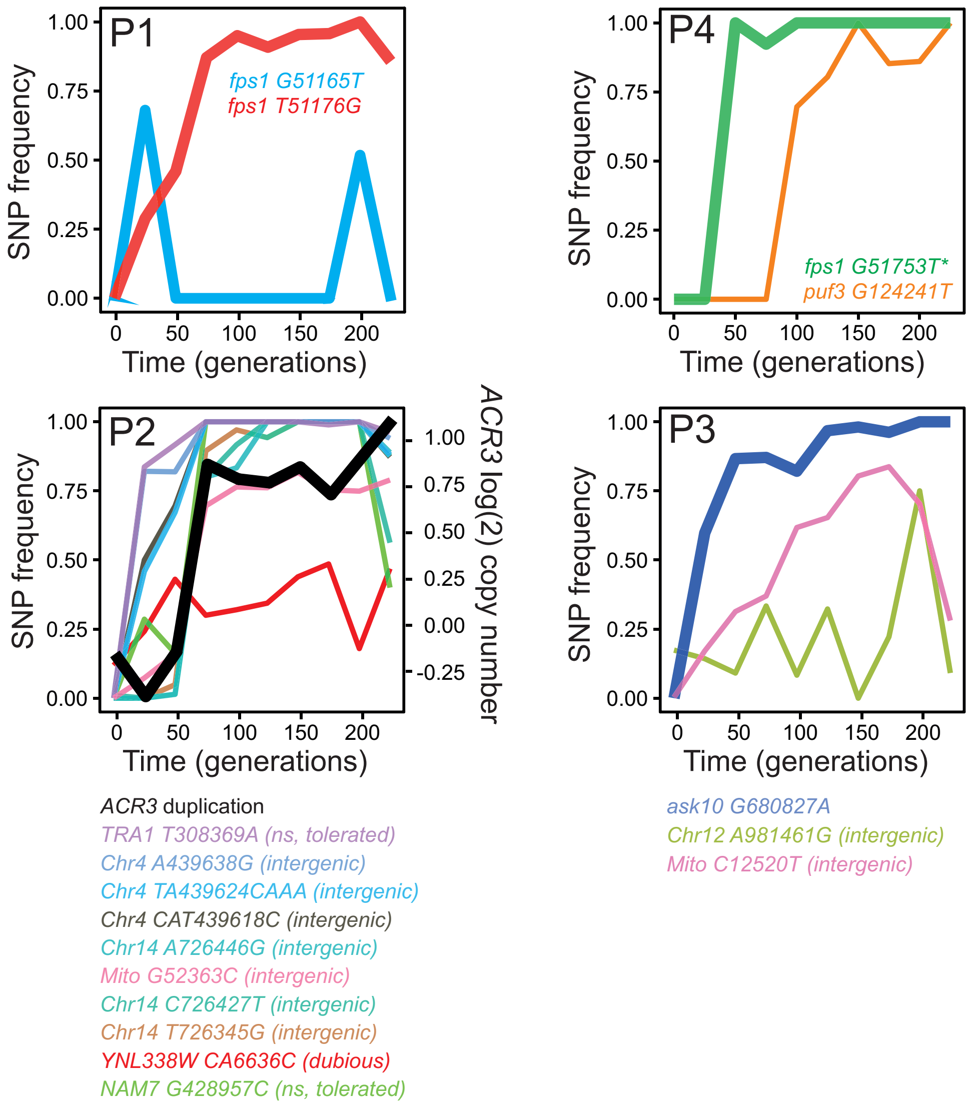


Figure S3 Gjuvsland et al

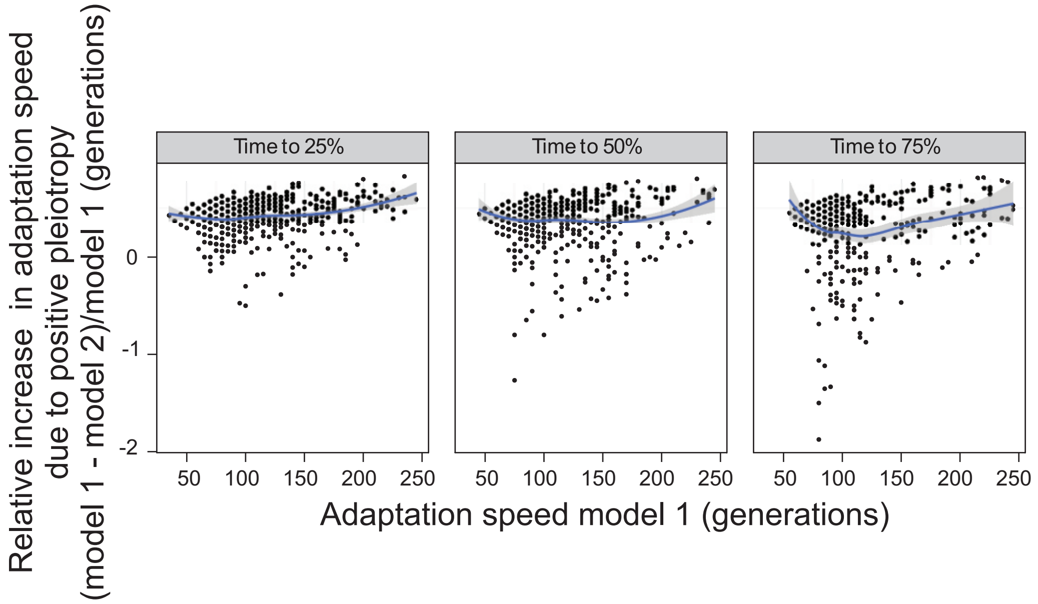


Figure S4 Gjuvsland et al

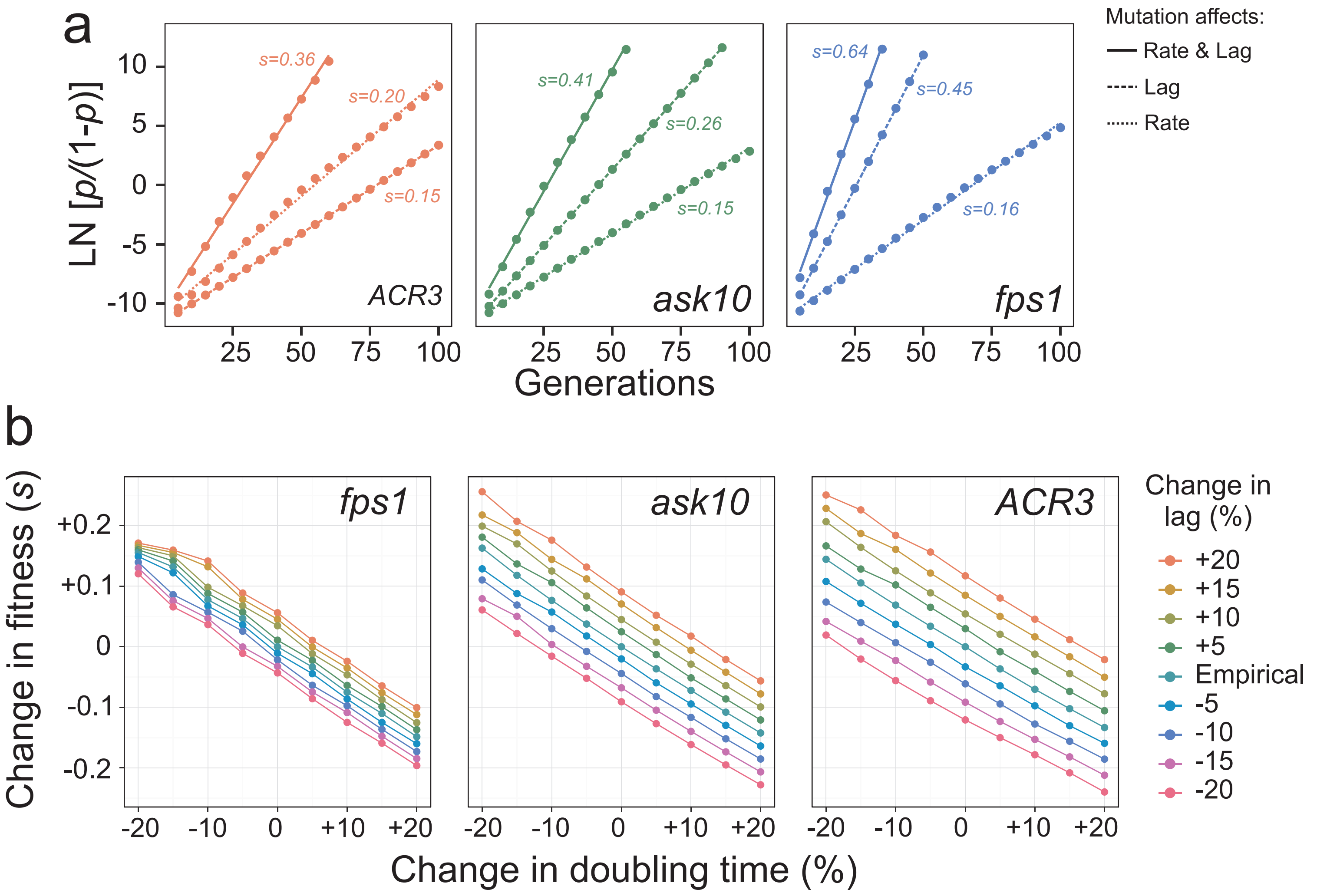


Figure S5 Gjuvsland et al

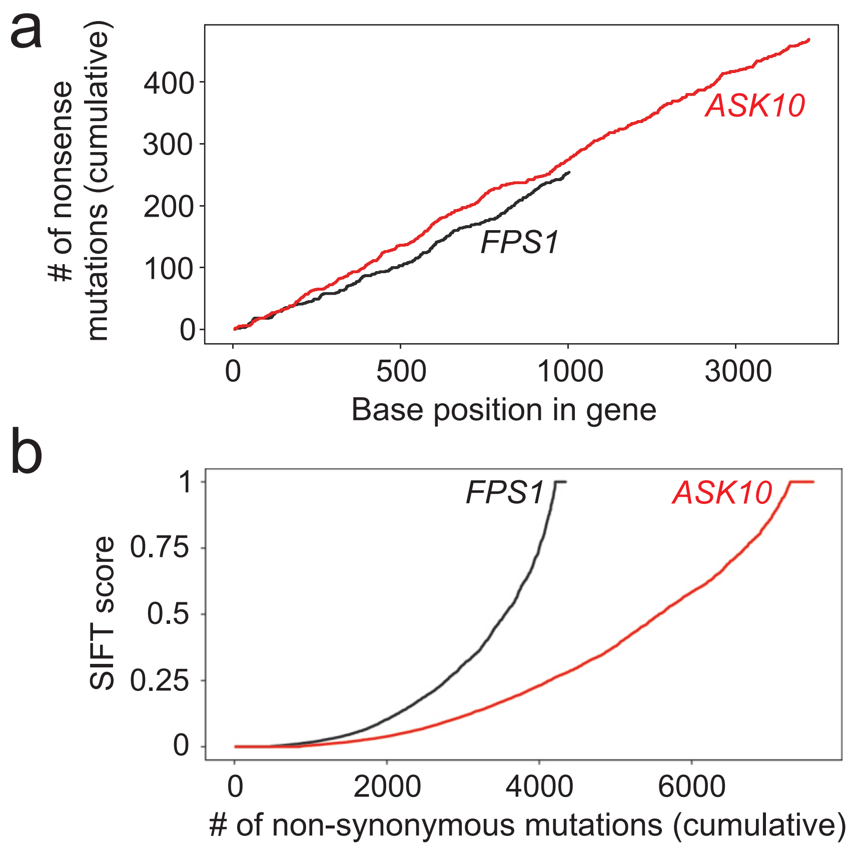


Figure S6 Gjuvsland et al

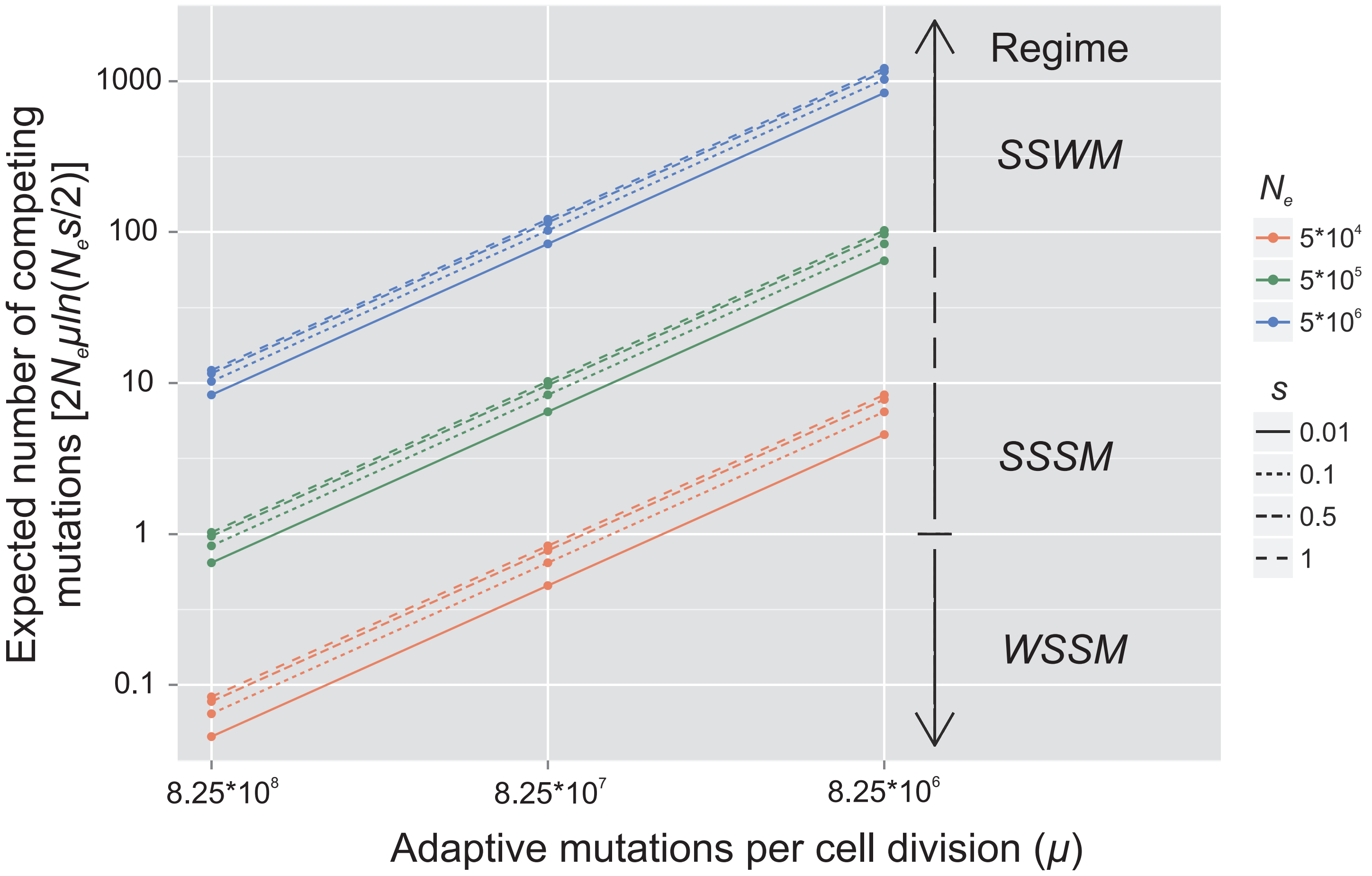


Figure S7 Gjuvsland et al

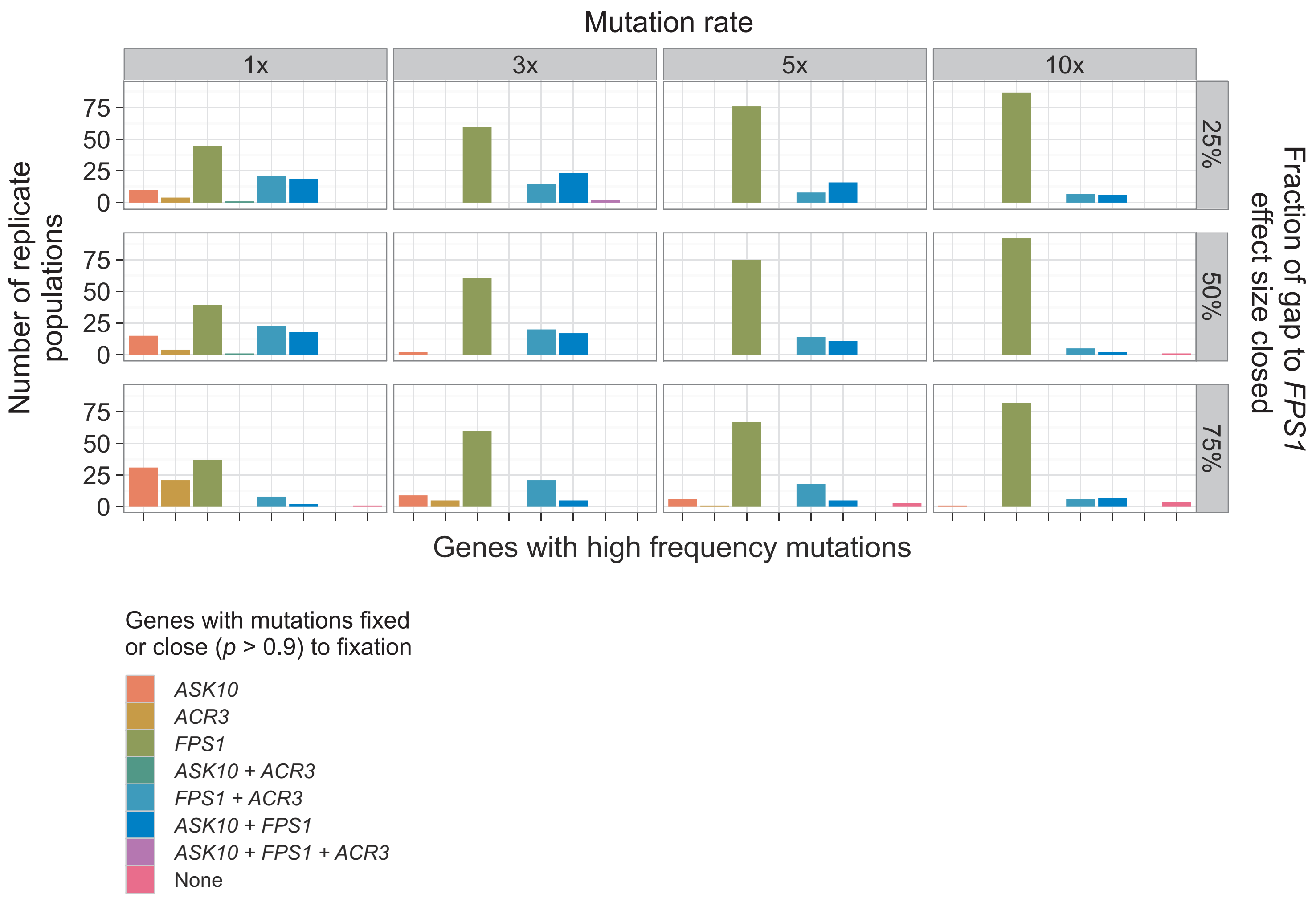


# Figure S8 Gjuvsland et al

**Appendix Table S1 Environmental challenges in selection experiments**

| Challenge (agent added) | Concentration |
|---|---|
| $NiCl_2$ | 0.5mM |
| $NaAsO_2$ | 5mM |
| CsCl | 0.185M |
| $CdCl_2$ | 0.2mM |
| $MgCl_2$ | 0.660M |
| Cycloheximide | 37.5ng/mL |
| $K_2TeO_3$ | 0.25mM |
| RbCl | 1.5M |
| Ethanol | 8% |
| NaCl | 1.4M |
| $AlCl_3$ | 0.4mg/mL |
| $CuCl_2$ | 1mM |
| Caffeine | 2.25mg/mL |
| $MnCl_2$ | 15mM |
| LiCl | 0.3M |
| Paraquat | 0.5mg/mL |
| $PbNO_3$ | 0.5mM |
| KCl | 2.28M |
| $CoCl_2$ | 0.02mM |

**Appendix Table S2 SNPs increasing in frequency during As(III) adaptation**.

| Population | Gene | Type | Function | Chromosome | Mutated position | Reads | | | |
|---|---|---|---|---|---|---|---|---|---|
| | | | | | | **Ref, founder** | **[illegible], founder** | **Ref, As(III)** | **[illegible], As(III)** |
| 4 | *FPS1** | Stop | Aquaglyceroporin | XII | G51753T | 22 | 0 | 1 | 54 |
| 3 | *ASK10* | Stop | Activator of Fps1 | VII | G680827A | 6 | 0 | 4 | 43 |
| 1 | *FPS1* | Nonsynonymous | Aquaglyceroporin | XII | T51176G | 26 | 0 | 29 | 25 |
| 1 | *FPS1* | Nonsynonymous | Aquaglyceroporin | XII | G51165T | 24 | 0 | 32 | 24 |
| 1 | *MDM31* | Promoter | Mitochondrial morphology | VIII | T490555A | 21 | 0 | 31 | 16 |
| 1 | *USB1* | Nonsynonymous | Unknown | XII | C407994A | 14 | 1 | 37 | 17 |
| 1 | *PTC7* | Promoter | Protein phosphatase | VIII | A250927G | 18 | 1 | 12 | 10 |
| 2 | *TCB3* | Nonsynonymous | Lipid-binding ER protein | XIII | A127923G | 14 | 0 | 14 | 11 |
| 1 | *PUF2* | Synonymous | mRNA stability regulator | XVI | A651310G | 23 | 1 | 37 | 12 |
| 3 | *Q0085; Q0081* | Intergenic | - | M | C28172G | 27 | 2 | 29 | 14 |
| 2 | *QRI7* | Nonsynonymous | Mitochondrial tRNA modifier | IV | C273698G | 16 | 2 | 13 | 13 |

SNPs with a large increase in frequency in As[III] adapting populations relative the founder. Reads denote number of reads containing either the reference genome variant (denoted ref) or the derived variant, in founder and As[III] adapted populations respectively. Mutated position gives the chromosomal coordinate for the nucleotide position affected. * = reconstructed *FPS1* mutation. M = Mitochondrial.

**Appendix Table S3 Copy Number Variations (CNVs) increasing in frequency during As[III] adaptation**

| Population | Genes in region | Type | Chromosome | Start (kb) | End (kb) | $Log_2$ coverage increase |
|---|---|---|---|---|---|---|
| 1 | *YAT1* | Amp | I | 191.3 | 192.1 | 1.1 |
| 1 | *HKR1* | Amp | IV | 1307.7 | 1308.4 | 0.9 |
| 1 | *YER152C, YER152W-A, PET122* | Amp | V | 473.6 | 474.4 | 0.8 |
| 1 | *PHO4* | Amp | VI | 225.1 | 225.8 | 0.9 |
| 1 | *SCS3* | Amp | VII | 271.3 | 272.1 | 1.2 |
| 2 | *SWH1* | Amp | I | 192.9 | 193.6 | 0.8 |
| 2 | *YBL113C* | Amp | II | 1.7 | 2.4 | 0.7 |
| 2 | *YCL041C, GLK1, YCL042W* | Amp | III | 50.6 | 51.3 | 0.7 |
| 2 | *PHO4* | Amp | VI | 225.1 | 225.8 | 0.9 |
| 2 | *YHL049C, YHL050C* | Amp | VIII | 3.2 | 3.9 | 0.7 |
| 2 | *YML133W-A, YML133C* | Amp | XIII | 1.4 | 2.6 | 0.7 |
| 2 | *AIF1, YNR062C, MNT4, YNR075C-A, YNR065C, YNR064C, BIO3-5, DSE4, HXT17, PDR18 YNR061C YNR073C, COS10, FRE4, BSC5 YNR068C, PAU6, YNR063W, YNR066C, HOL1, YNR071C* | Del | XIV | 726.4 | 782 | -3.3 |
| 2 | *YNR077C* | Del | XIV | 782.7 | 784.2 | -1.2 |
| 2 | *WHI5, LPX1* | Amp | XV | 480.3 | 481.2 | 0.88 |
| 2 | *RPO26, RPC82, ACR1-3, BSP1, YPR169W-A, YPR174C, PRP4, YPR170W-B, OPT2, BET2, SGE1, SEC23, VPS4, QCR2, DPM1, AOS1, YPR196W, SKI3, AQY1, GDB1, MLC2, YPR172W, YPR195C, YPR170W-A, DPB2, YPR197C, HPA2, SMX3, YPR170C, PZF1, YPR177C, HDA3, ATG13* | Amp | XVI | 881.2 | 950.7 | 0.9 |
| 3 | *YAT1* | Amp | I | 190.8 | 192 | 1.1 |
| 3 | *GLK1, YCL042W* | Amp | III | 50.7 | 51.7 | 0.8 |
| 3 | *SOL2* | Amp | III | 246.9 | 247.6 | 0.8 |
| 3 | *YDR261C-D* | Del | IV | 986.9 | 987.5 | -0.7 |
| 3 | *YER152C, YER152W-A, PET122* | Amp | V | 473.6 | 474.8 | 0.9 |
| 3 | *PHO4* | Amp | VI | 225.1 | 225.9 | 0.8 |
| 3 | *SCS3* | Amp | VII | 271.2 | 271.8 | 1 |

| | | | | | | |
|---|---|---|---|---|---|---|
| 3 | *TOP3, YLR235C* | Amp | XII | 611 | 611.7 | 0.9 |
| 3 | *LPX1* | Amp | XV | 480.3 | 481.3 | 0.9 |
| 3 | *PUT4* | Amp | XV | 987.2 | 987.9 | 0.8 |
| 4 | *SCS3* | Amp | VII | 271.2 | 271.9 | 0.8 |
| 4 | *YLR302C* | Amp | XII | 732.3 | 733 | 1.2 |

Copy number variations were defined as regions with an average change in $\log_2$ read coverage change > 0.5 comparing As[III] adapted to founder populations. Amp/Del = Amplification or deletion of chromosome segment. Start and end gives estimated chromosomal coordinates for the boundaries of the CNV.
