# Supplementary material for: Disentangling genetic and epigenetic determinants of ultrafast adaptation
Source: Mol Syst Biol. 2016 Dec 15;12(12):892. doi: 10.15252/msb.20166951 (PMC5199126; doi:10.15252/msb.20166951)
Supplement: Supplementary file 2 — Expaned View Figures PDF [file MSB-12-892-s002.pdf]

Expanded View Figures

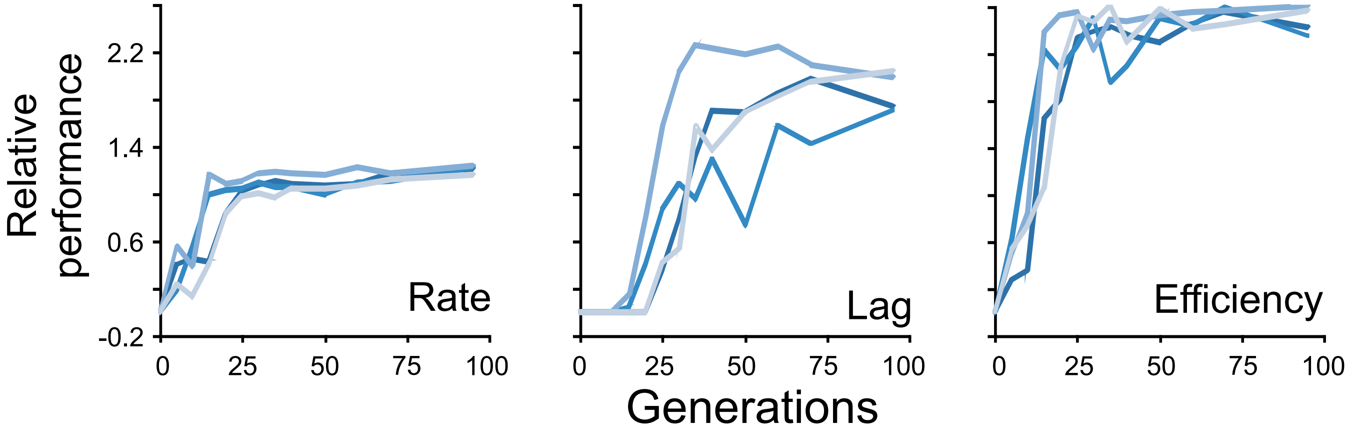

Figure EV1. Ultrafast arsenic adaptation is unlikely to be driven by standing genetic variation.
As(III) adaptations were repeated, but starting from four separate founder populations, each derived from different (single) founder clones (P5–P8). The probability of standing variants being present at the onset of selection in all four populations is small (see Materials and Methods). Samples were extracted at the end of each batch cycle, that is every 5th generation, stored as a frozen fossil record, revived in fresh medium and cultivated in 5 mM As(III) at high replication (n = 10). Fitness components were extracted and analysed as for Fig 1B.

Source data are available online for this figure.

 © 2016 The Authors

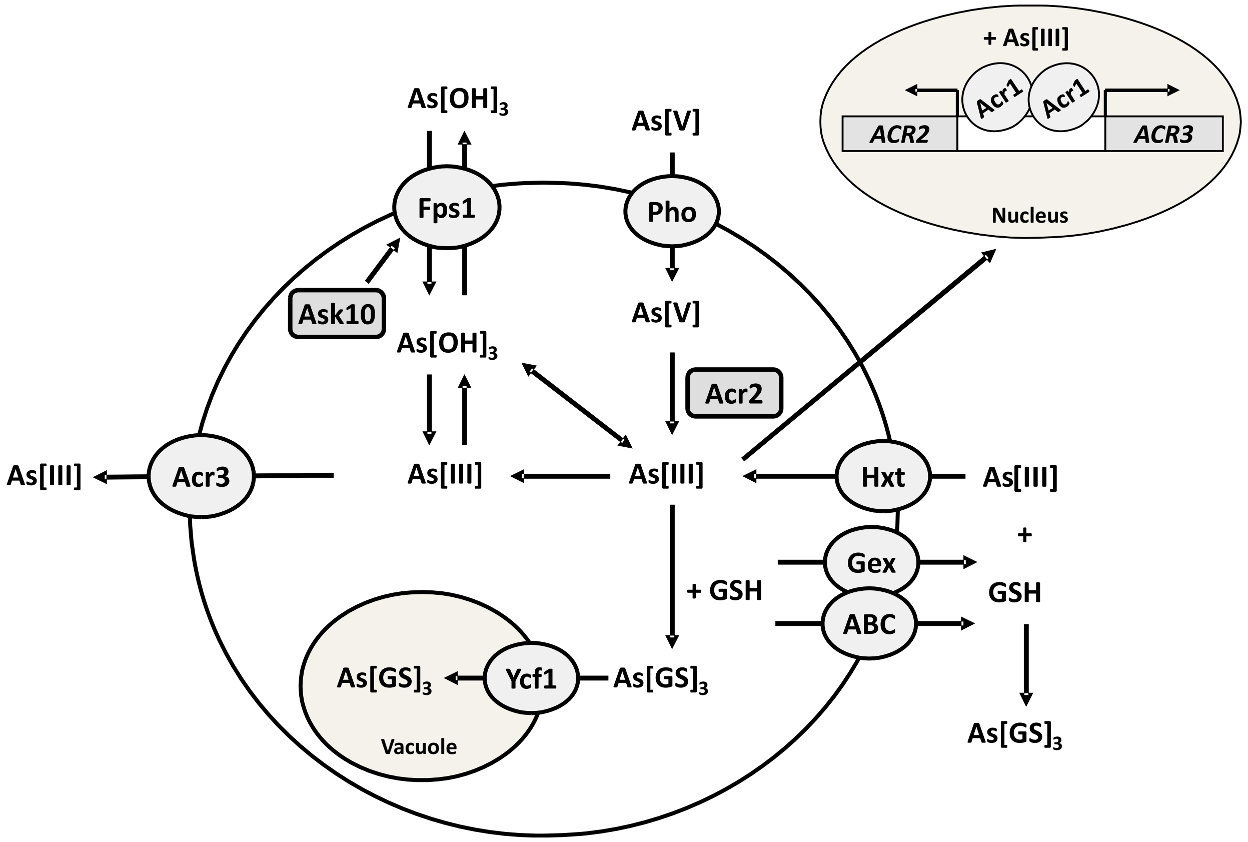

Figure EV2. Model of arsenic metabolism in yeast.

Pentavalent arsenate As(V) enters yeast cells via phosphate transporters (Pho) (Bun-ya *et al*, 1996; Yompakdee *et al*, 1996). The arsenate reductase Acr2 reduces cytosolic As(V) to trivalent arsenite As(III) (Mukhopadhyay & Rosen, 1998; Mukhopadhyay *et al*, 2000) followed by export by the arsenite permease Acr3 (Wysocki *et al*, 1997), probably in the form $As(OH)_2O^-$, or by import into the vacuole by the ABC transporter Ycf1 as the glutathione conjugate $As(GS)_3$ (Ghosh *et al*, 1999). As(III) enters cells through the aquaglyceroporin Fps1 (Wysocki *et al*, 2001) in the form $As(OH)_3$ and through hexose permeases (Hxt) (Liu *et al*, 2004). As(III) can also be extruded via Fps1 down the concentration gradient (Bienert *et al*, 2008; Maciaszczyk-Dziubinska *et al*, 2010). *S. cerevisiae* increases glutathione (GSH) biosynthesis in response to As(III) exposure for intracellular chelation and protection (Thorsen *et al*, 2007; Talemi *et al*, 2014). GSH is also exported via plasma membrane localized ABC transporters (ABC) and specific GSH export proteins (Gex) for extracellular As(III) chelation/detoxification(Thorsen *et al*, 2012). Acr1 (also called Yap8 or Arr1) is a nuclear transcription factor that senses As(III) and activates transcription of *ACR2* and *ACR3* (Wysocki *et al*, 2004). Ask10 (also called Rgc2) is a positive regulator of Fps1 (Beese *et al*, 2009). For recent overviews of arsenic uptake and detoxification pathways in yeast, see (Wysocki & Tamas, 2010; Wysocki & Tamás, 2011; Talemi *et al*, 2014).

© 2016 The Authors

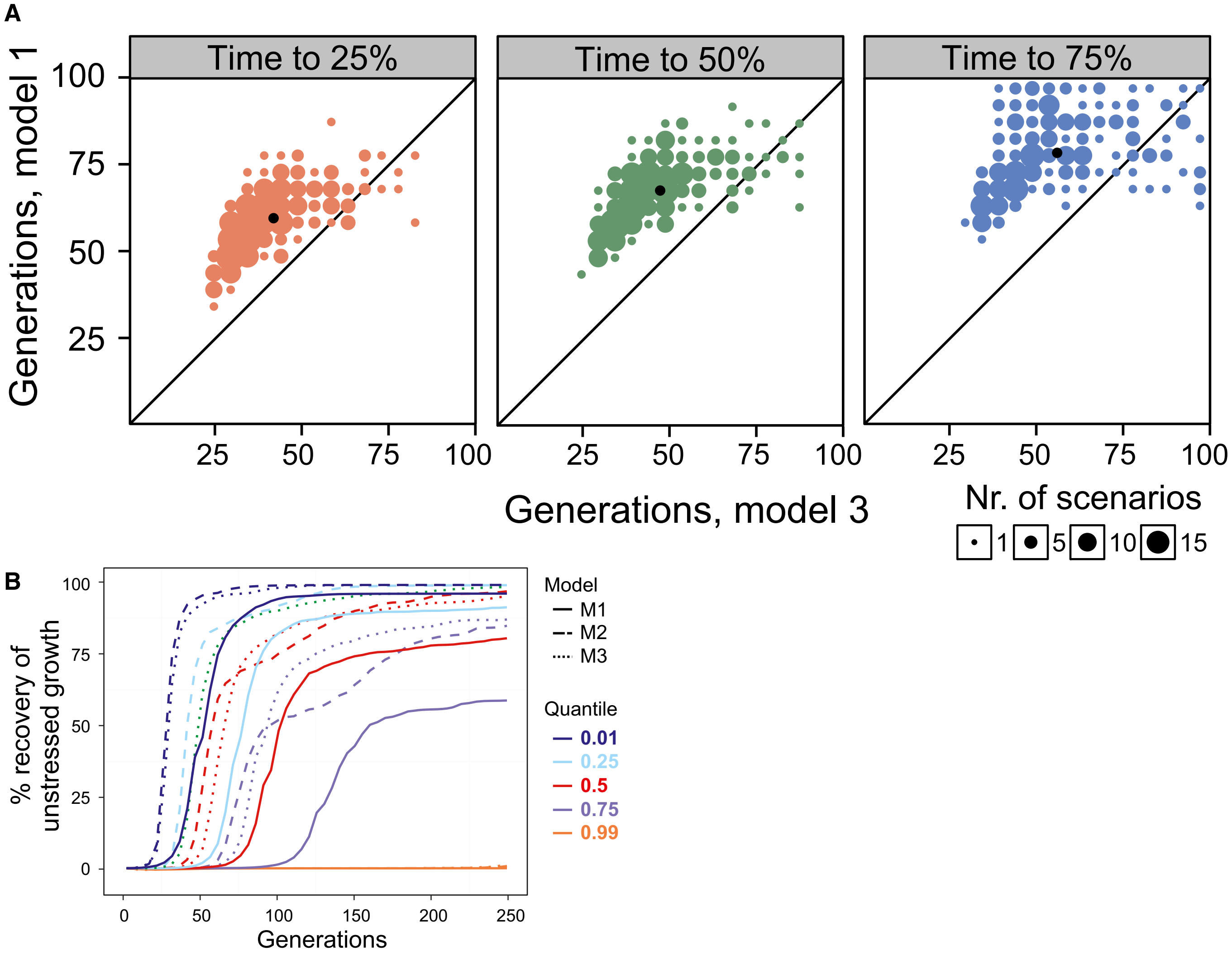

Figure EV3. Positive pleiotropy accelerates adaptation.

A, B Simulated populations (n = 500) adapting to As(III) in a model mimicking the experimental set-up. Each population has a distinct set of mutation parameters driving intermediate to fast adaptation, and each cell has a genotype–phenotype map where mutations affect cell division time (doubling time), time to the first cell division (growth lag), or both. Phenotypes were extracted every 5th cell division, population means were estimated and expressed as the degree to which the population had recovered unstressed performance (doubling time), that is 0 and 100% correspond to WT performance in presence and absence of arsenic respectively. (A) Contrasting models in which mutations affect only rate (M1, y-axis) and both rate and lag with random strength and direction (M3, x-axis). Number of cell divisions to recover 25% (left panel), 50% (middle panel) and 75% (right panel) of unstressed doubling time is shown. Only the 204 fastest scenarios, recovering 75% of unstressed performance in ≤ 100 cell divisions, are displayed. Dot size = number of populations in that position. Black dot = median population. (B) Visual comparison of populations adapting to arsenic in models where mutations affect only rate (M1, full lines), both rate and lag with similar effect size and direction (M2, dashed lines) and both rate and lag, but with random effect size and direction (M3, dotted lines). Quantiles corresponding to the fastest adapting 1, 25, 50, 75 and 99% of populations are shown.

© 2016 The Authors

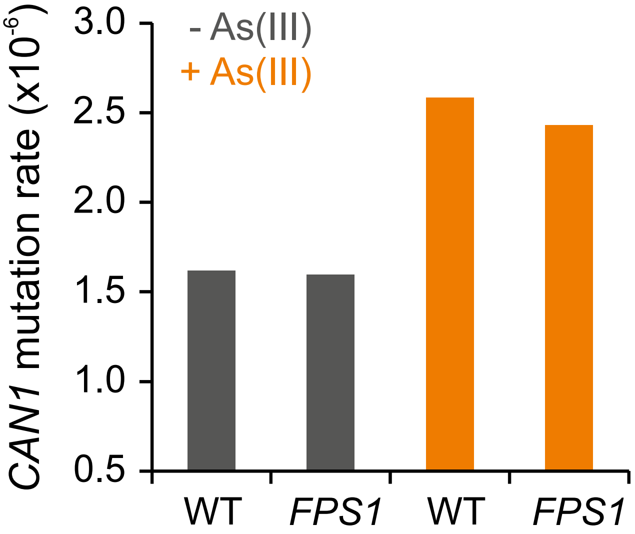

Figure EV4. Mutation rates are near basal at *CAN1* during As(III) exposure.

Loss-of-function mutation rate at the *CAN1* locus, measured by fluctuation assay, in founder (WT) and As(III) adapted (*FPS1*) cells, with and without As(III) exposure. *y*-axis = the *CAN1* loss-of function mutation rate per *CAN1* locus and cell division.

Source data are available online for this figure.

© 2016 The Authors
